# Editorial: Assessment of intraoperative image technologies to optimize clinical outcomes in neurosurgical oncology

**DOI:** 10.3389/fonc.2023.1202240

**Published:** 2023-05-04

**Authors:** Sergio García-García, Julius Höhne, Rafael Martinez-Pérez, Santiago Cepeda

**Affiliations:** ^1^ Neurosurgery Department, Hospital Universitario Río Hortega, Valladolid, Spain; ^2^ Neurosurgery Department, University Medical Center Regensburg, Regensburg, Germany; ^3^ Neurosurgery Department, Geisinger Medical Center, Danville, PA, United States

**Keywords:** intraoperative imaging, neurosurgical oncology, intraoperative magnetic resonance, intraoperative ultrasounds, photodynamic therapy, optical coherence tomography, confocal laser imaging, cost-effectiveness

During the recent decades, neurosurgery and neurosurgical oncology have undergone a significant technological revolution, with new devices and applications being introduced to increase surgical success rates and ensure patient safety. One critical innovation in this field is intraoperative imaging, which has played a paramount role in providing reliable feedback to surgeons during surgeries. From neuronavigation to augmented reality, a broad range of intraoperative imaging techniques are currently available, each promising to overcome the limitations of its predecessors. However, the rapid pace of technological progress has prevented a thorough evaluation of the actual benefits of these new technologies, leading to a lack of robust evidence to support their adoption. Indeed, the economic perspective of these advancements and assumed improvements have been largely disregarded, further hindering the offer of comprehensive recommendations to health systems worldwide.

## Increasing the extent of resection in neurosurgical oncology

In neurosurgical oncology, recent research has focused on two main objectives: maximizing the extent of tumor resection while preserving functionality and studying tumoral and peritumoral samples in the intraoperative setting (1, Restelli et al.). This Research Topic highlights the advancements in intraoperative imaging techniques contributing to these essential goals.

In the quest to enhance tumor resection, several studies have explored the use of intraoperative ultrasound (IoUS) and magnetic resonance imaging (ioMRI) (1–7). Wang et al. demonstrated the effectiveness of IoUS in recurrent glioma surgery, resulting in reduced residual tumor volume, improved postoperative outcomes, and fewer recurrences. Concurrently, Becerra et al. found that 1.5-T high-field ioMRI was a safe and dependable tool in pediatric neuro-oncology surgeries, maximizing tumor resection without increasing neurological deficits or complications.

Integrating diffusion tensor imaging (DTI) tractography with neuronavigation, as explored by Shi et al., has shown promise in preserving visual function during tumor resection in the optic radiation area. Combining these techniques led to better visual outcomes and the identification of clinical factors impacting patients’ visual function and quality of life.

A new and burgeoning field in glioma surgery involves the use of 5-ALA, not as a surgical guidance tool to enhance extent of resection but as a therapeutic adjuvant. Ferres et al. observed a significant reduction on glioma recurrence within the first centimeter from the surface of surgical cavity in a cohort of patients undergoing 5-ALA guided surgery compared to those operated without it. Their findings, supported by previous evidence (8, 9), lead authors to recommend intensifying research efforts in this promising field (Ferres et al.).

## Intraoperative approach to histological sample analysis

In parallel with the advancements in tumor resection techniques, researchers have also made strides in studying tumoral and peritumoral samples in the intraoperative setting. Restelli et al. conducted a comprehensive review of sodium fluorescein-based confocal laser imaging using the CONVIVO system, highlighting its promising diagnostic performance compared to standard histopathology methods. Nonetheless, further optimization of sodium fluorescein protocols and larger clinical trials are necessary to establish its position in routine clinical practice.

The potential of optical coherence tomography (OCT) for detecting peritumoral white matter damage and residual tumor detection has been investigated by Achkasova et al. and Kuppler et al. Achkasova et al. found that visual assessment of structural OCT images and color-coded maps enabled differentiation of tissue types, with color-coded maps exhibiting higher diagnostic accuracy. Kuppler et al. reported that contactless *in vivo* OCT scanning achieved high accuracy for residual tumor detection, supporting *ex vivo* OCT brain tumor scanning and complementing existing intraoperative techniques.

## Intraoperative imaging devices in endoscopic skull base surgery

While advancements in optics, lighting, and imaging displays have greatly improved the field of endoscopic skull base surgery, the adoption of surgical innovations used in open surgery has been limited. Recent advancements in probe sizes and image reconstruction algorithms have increased the use of IoUS in endoscopic skull base surgery (10, 11). End-firing and side-firing probes enhance depth assessment and anatomical real-time guidance during surgery. In this Research Topic, Baker et al. illustrated the utility and potential benefits of side-firing IoUS in endoscopic surgeries for clival chordomas and neuroendocrine pituitary tumors (Baker et al.). Through their research, Baker et al. have demonstrated that the use of IoUS in endoscopic surgery improves surgeon´s judgement of extent of resection. Additionally, this technique demonstrated reduced operative time and the decreased incidence of postoperative endocrine deficits.

## Cost effectiveness evidence for intraoperative image technologies

Literature regarding economic evaluation of surgical innovations in neurosurgery is scarce (2, 12). Previous studies have not provided conclusive evidence for a positive correlation between the cost of implementing modern technologies and their clinical benefits. Mosteiro et al. conducted a comparative cost-effectivenes study of intraoperative magnetic resonance (iMR) and IoUS in glioma surgery. Authors found that although iMR might be more expensive and time-consuming, it yielded better clinical outcomes in terms of extent of resection and postoperative performance status. As a result, iMR was found to be cost-effective. However, efforts should be addressed to thoroughly evaluate surgical technological advancements from a clinical and economic perspective, centered on patient care and on the respective social context.

## Conclusion

This collection of ten articles offers new insights on surgical innovations applied to neuroncology: new applications of available devices; cutting-edge technologies; clinical series evaluating the benefits of state-of-the-art intraoperative imaging and a necessary study on cost-effectiveness assessment. Moving forward, it will be essential to conduct rigorous clinical trials to validate these techniques and establish standardized protocols for their adoption in settings where their benefit might be optimal. As the field continues to evolve, the insights and findings presented in this collection will serve as an important foundation for further advancements in surgical innovation.

## Author contributions

SG-G and SC designed and wrote the draft of this editorial which was posteriorly reviewed by JH and RM-P. All authors approved the final version of the manuscript.
